# Home-Made Membraneless Vaporization Gas-Liquid Separator for Colorimetric Determination of Ethanol in Alcoholic Beverages

**DOI:** 10.1155/2022/7346253

**Published:** 2022-03-31

**Authors:** Vanpaseuth Phouthavong, Hayato Inoue, Kesiny Phomkeona, Vanseng Chounlamany

**Affiliations:** ^1^Department of Chemistry, Faculty of Natural Sciences, National University of Laos, P.O. Box 7322, Vientiane, Laos; ^2^Department of Environmental and Life Sciences, Toyohashi University of Technology, 1-1 Hibarigaoka Tempaku-cho, Toyohashi, Aichi 441-8580, Japan

## Abstract

This work utilized the simplicity of a so-called membraneless vaporization (MBL-VP) unit as a gas separator for the colorimetric determination of ethanol in alcoholic beverages. A beverage sample with a volume of 1 mL was directly injected into a small container which was hung from a lid inside a closed 40 mL reused glass bottle without pretreatment such as distillation. An acidified potassium dichromate (Cr_2_O_7_^2−^) acceptor solution, preadded to the glass bottle, was reduced to Chromium (III) ion by the diffusion of vaporized ethanol from the sample. After 5 min, the absorbing solution was collected for colorimetric detection at 590 nm. The unit manually quantifies ethanol in the range 1.0–90% (v/v) with satisfactory interday precision but without matrix effect (recovery 89−109%). The method was validated with the conventional distillation/pycnometer method which showed no significant difference of ethanol contents between those two methods and the declared values of 12 alcoholic beverages, indicating sufficient accuracy. Analyses of alcoholic beverages using this method were successful with benefits of simplicity, cheapness, and less energy consumption.

## 1. Introduction

Quantification of ethanol, a major compound present in every alcoholic drink, is important for quality control of the beverages. The well-known conventional physical method for quantifying ethanol degree in beverages is the use of a hydrometer or pycnometer [1–3]. Other official chemical methods are based on the oxidation of ethanol by dichromate (Cr_2_O_7_^2−^) producing Chromium (III) (Cr^3+^) ion with subsequent monitoring by titration or colorimetric detection [1, 4], which are reliable but require a high sample volume, reagents, and energy consumption. Reliable and less sample consumed analysis of ethanol can be accomplished using instrumental techniques such as gas chromatography [5–7], liquid chromatography [8, 9], infrared spectrometry (IR) [10], Raman spectrometry [11, 12], nuclear magnetic resonance spectrometry (NMR) [13], and mass spectrometry [14], but the instruments are expensive and require a skillful operator. Alternatively, using sensor-based methods is another choice [15–19], but expensive and specific chemicals are consumed for complicated preparation of the particular sensors. Colorimetric methods using ultraviolet (UV)/visible spectrophotometers were widely applied for ethanol quantification due to their ease of use and less expensiveness, but sample preparation to separate ethanol from matrix is still required. Several approaches have recently been developed to replace the old-fashioned energy-consumed distillation method to comply with the green analytical chemistry strategies [20]. These works were successfully developed for the analysis of ethanol in beverages by minimizing sample size and reagent without sample pretreatment in a lab-on-chip device [21], using a smartphone camera [22–24] or a desktop scanner [25] as a miniaturized detection tool, and using nontoxic reagent with simple electroconductivity detection [26].

The volatility of ethanol was employed in the so-called gas diffusion (GD) techniques for gas-liquid separation incorporated with flow-based systems. In the GD techniques, the ethanol donor and the acceptor stream, a Cr_2_O_7_^2−^ solution in most cases, can be separated using a planar hydrophobic porous membrane [27–30] and tubular membrane [31], the membrane with a headspace on the donor stream, called pervaporation [32–35], or without membrane, called membraneless GD [36]. In those three GD techniques, vaporized ethanol diffused from the donor to the acceptor for chemical reaction to take place. Then, the reaction product zone was propelled to a detector in a flow analysis system. In this manner, high sample throughput was achieved to determine ethanol in various drink samples. Recently, a permeable membrane was fabricated on a Cr_2_O_7_^2−^-preloaded melamine foam as a novel ethanol indicator for preservation of baby mangoes [37]. The membraneless GD (abbreviated as MGD) was firstly proposed by Choengchan et al. to overcome the drawbacks of clogging and frequent replacement of membrane when using GD or PV [36]. Inside the MGD, unit donor and acceptor channels were designed to be in parallel covered with headspace. Mass transfer of vaporized ethanol from donor to the acceptor (Cr_2_O_7_^2−^) occurred in the headspace. Another design of the nonmembrane gas-liquid separator in flow-based operation was developed, which is not only improved in analysis of drink samples [38, 39], but applied in analysis of environmental samples [40–43]. However, an equal flow rate of donor and acceptor streams is required to avoid overflow and contamination when using the MGD unit. Application of mass transfer of ethanol in a headspace was also proposed as a single-drop headspace microextraction [44]. Vaporized ethanol diffused in a Cr_2_O_7_^2−^ solution drop that was positioned in the inlet of the syringe. The operation was automated with low reagent consumption but stabilization of the single-drop was crucial. Sereenonchai et al. [45] proposed another nonmembrane gas separator named membraneless vaporization unit (MBL-VP unit) to direct analysis of solid samples in a flow analysis system. Solid samples were manually introduced into a replaceable vial surrounded by an acceptor channel. Gaseous species in the sample was released by injection of a reagent. The MBL-VP unit was applied in analyses of carbonate in calcium supplements [45] and cements [46], monitoring of sugar, color, and dissolved carbon dioxide in beverage production [47, 48]. In 2014, a simpler MBL-VP unit was designed and presented by Kookarinrat et al. for entirely manual operation of carbonate determination in solid and liquid samples [49]. A replaceable glass sample vial was placed at the bottom of the glass jar, surrounded by an acceptor solution, for fabrication. Recently, gas-liquid separation can be carried out using simple and low-cost apparatus such as microtubes and centrifuge tubes for determination of methanol in biodiesel [50] and ethanol in fermented sugarcane substrate [51]. These works showed high potential as alternative analysis tools with the benefits of simplicity and cheapness of the gas-liquid separator fabrication.

In this work, the simplicity and cheapness of the home-made MBL-VP unit were utilized as a gas separator which extended to the direct determination of ethanol in alcoholic beverages. Based on the high volatility of ethanol, alcoholic beverage samples were therefore directly introduced to the unit without pretreatment and the use of additional reagent. Dichromate oxidation was used for the chemical reaction between the vaporized ethanol and acceptor solution (acidic Cr_2_O_7_^2−^). Chromium (III) ion product was detected as absorbance at 590 nm, corresponding to ethanol content in the sample.

## 2. Experimental

### 2.1. Chemicals and Instruments

All chemicals were of analytical reagent (AR) grade and used without further purification. Absolute ethanol (99.9%, Merck, USA) was used as standard ethanol. Dilution of this standard with double distilled water was carried out to obtain the required concentrations in percentage by volume (% (v/v)) to prepare standard calibration curves. An acceptor solution, 0.2 mol L^−1^ potassium dichromate (K_2_Cr_2_O_7_, Fischer, Germany) in 4.0 mol L^−1^ sulfuric acid (H_2_SO_4_, Merck, USA), was prepared according to [32]. A magnetic stirrer (Stuart Scientific, UK) was used for stirring the sample during ethanol separation. Measurement of absorbance was performed using a UV-Visible spectrophotometer (CE1011-1000 Series, Cecil, England) equipped with a 1 cm LightPath glass cuvette. Apparatus for measuring specific gravity: a 25 mL borosilicate pycnometer (Thomas Scientific) and a 4-decimal digital balance (SECURA224–1S, Satorius, Germany) were used for method validation.

### 2.2. Beverage Samples

Twelve alcoholic beverages, including beer, wine, spirit, and cocktail, were obtained from local stores in Vientiane, Lao PDR. All samples were directly analysed using the proposed method without sample pretreatment.

### 2.3. Construction of the MBL-VP Unit for Ethanol Separation

**Figure 1:**
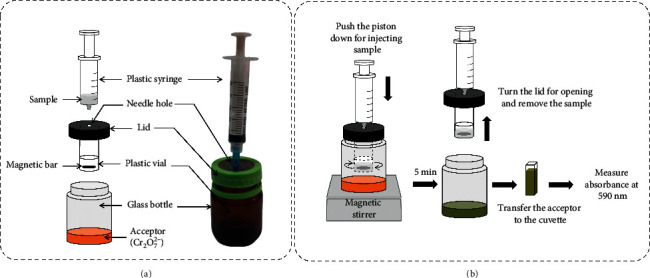
Construction (a) and brief operational procedure (b) of the MBL-VP unit for colorimetric determination of ethanol in beverages.

The MBL-VP unit for ethanol separation was simply fabricated, as shown in Figure 1(a). A reused fruit essence drink glass bottle (≈40 mL, BRAND'S®, Thailand) itself served as the acceptor container. On the center of the lid, there was a small hole drilled for inserting a needle for injection of beverage samples. Injection of the samples was carried out by using a disposable 5 mL plastic syringe (Nipro, Thailand). A 5 mL plastic sample vial containing a minimagnetic bar was hung from the lid, which was easily removable together in one-turn counterclockwise. This design aimed to remove residual ethanol vapour faster and stop the absorption of ethanol vapor into the acceptor when the optimal trapping time was reached. This is a slight modification from the previous simple jam jar apparatus where the sample container was placed on the bottom of the glass jar [49].

### 2.4. Operation of the MBL-VP Unit for Colorimetric Determination of Ethanol in Beverages

The brief operation is shown in Figure 1(b). Firstly, 2.0 mL of the acceptor solution was added to the acceptor container using a pipette. The container was tightly capped and placed on the magnetic stirrer. Secondly, 1 mL of the sample was graduated and injected using a plastic syringe into the sample vial. Vaporized ethanol present in the sample was trapped for 5 min. During the trapping time, the merging of ethanol into the orange acidic Cr_2_O_7_^2−^ solution occurred, resulting in the color change of the acceptor to green. After 5 min, stirring was stopped, and the acceptor container was immediately opened to remove the excess vaporized ethanol and stop the gas diffusion process. The acceptor solution was finally transferred to a glass cuvette for measuring its absorbance at 590 nm.

### 2.5. Optimization and Analytical Performance

To obtain the highest performance, parameters such as sample volume, vapor trapping time, and reaction time were optimized by univariation. In this work, optimal reagent concentrations reported by Ratanawimarnwong et al. [38] were employed. The volume of the acceptor was fixed at 2.0 mL since this amount was sufficient for single absorbance measurement. A standard ethanol solution (40% (v/v)) was used for all optimization studies. The optimal conditions were then employed to evaluate analytical performance of the proposed method such as linear range, detection and quantification limits, precision, and accuracy.

### 2.6. Method Validation

The proposed MBL-VP unit was validated using conventional distillation/pycnometry. Briefly, exact 200.0 mL of a beverage sample was graduated using a 200 mL volumetric flask and transferred to distillation apparatus. The distillation was carried out at 80°C until no more distillate was collected (approximately 35 min). After cooling, the distillate was diluted to 200.0 mL with double-distilled water in a 200 mL volumetric flask. The diluted ethanol was transferred to the preweighed pycnometer for measuring the specific gravity of the solution. The specific gravity was then converted to ethanol content in % (v/v) using the specific gravity table of the water-ethanol mixtures at 20°C [2, 3].

## 3. Results and Discussion

### 3.1. Optimization Studies

#### 3.1.1. Sample Volume

**Figure 2:**
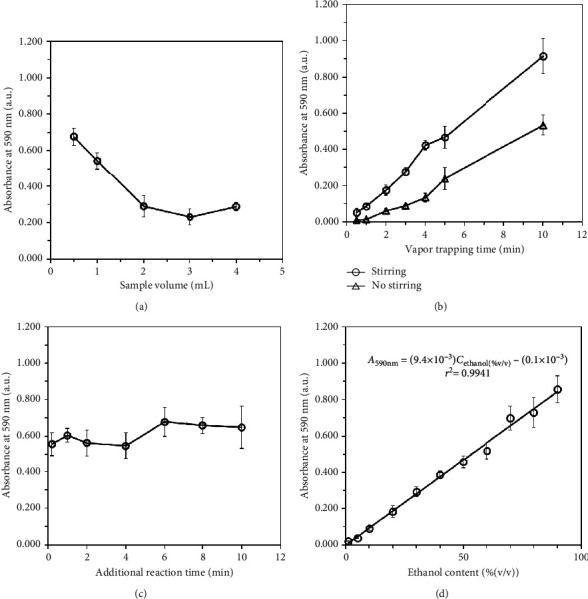
Optimization results of the effect of sample volume (a), vapor trapping time (b), additional reaction time before absorbance measurement (c), and linearity of the proposed MBL-VP unit for colorimetric determination of ethanol (d).

This parameter was studied in order to evaluate the effect of sample volume or donor volume on the evaporation of ethanol. For a fixed amount of the analyte, evaporation of the volatile species could vary from smaller to larger donor volume, which affects the mass transfer of the analyte between the liquid donor phase and the headspace. In this work, a sample volume in the range of 0.5–4 mL containing a fixed mole of ethanol was studied. As shown in Figure 2(a), using a 0.5 mL sample gave the highest absorbance, indicating that the highest evaporated ethanol was obtained in the studied range. Increasing sample volume from 0.5 to 2 mL resulted in a dramatic decrease in absorbance and remained unchanged up to 4 mL. At lower sample volume, the solution in the container was shallower, and vaporization of ethanol was much easier, facilitating the quicker mass transfer. It, therefore, yielded higher absorbance than the larger ones by using a fixed amount of ethanol. In conclusion, the lower donor volume, the easier evaporation of ethanol. However, considering the method sensitivity when low-ethanol samples are analysed, a larger sample volume is required in order to obtain more amount of ethanol in the donor phase. Therefore, a sample volume of 1 mL was considered for further uses throughout the experiments.

#### 3.1.2. Vapor Trapping Time

After introducing a sample to the donor vial, a fixed period of time is required for trapping the vaporized ethanol which is subsequently absorbed into the acceptor solution for oxidation to take place. The optimal trapping time was therefore studied to obtain sufficient method sensitivity. In this work, trapping time was immediately counted after injection of the sample into the unit until the lid was opened. The results in Figure 2(b) indicated that increasing trapping time from 0.5 to 10 min higher absorbance was observed since more vaporized ethanol was donated to the acidic dichromate acceptor. To facilitate the mass transfer, the sample was simultaneously stirred using a magnetic stirrer. As obviously seen in Figure 2(b), a trapping time of 10 min was required to obtain absorbance of 0.5, but it was shortened to 5 min when stirring was introduced. Additionally, the absorbance was doubled when trapping time was increased from 5 to 10 min. Therefore, the vaporization of ethanol was greatly improved by stirring. It was found that trapping time of 5 min with continuous stirring provided satisfactory analysis time with sufficient sensitivity. A trapping time of 5 min was consequently selected for further experiments. Since the vaporization of ethanol did not reach saturation within 10 min with the average increasing rate of absorbance of approximately 0.09 a.u./min, it therefore critically affected precision of the manual operation. To avoid large variation of the measurement, immediate opening of the lid was strongly recommended when 5 min trapping time was reached. By this manner, vaporization and diffusion of ethanol into the acceptor solution could be manually controlled. In this work, the sample container was hung to the lid of the MBL-VP unit, allowing quick removal of the sample and residual vapor when one-turned opening the lid. Thus, a large measurement variation was not encountered when this manual operation was used.

#### 3.1.3. Additional Reaction Time

After the lid was opened and before measuring absorbance, the appropriate additional waiting time might be required to completely oxidize the absorbed ethanol in the acidic Cr_2_O_7_^2^⁻acceptor solution. This parameter affected to the method sensitivity and analysis time. The results (Figure 2(c)) indicated that no significant change in absorbance was found up to 10 min compared to the first data point where immediate absorbance measurement was performed (no additional waiting time). This result revealed that the trapped vaporized ethanol was completely absorbed into the acceptor and rapidly reduced Cr_2_O_7_^2−^ (aq) to Cr^3+^ (aq) within the period of 5 min trapping time. Therefore, no additional waiting time was required, and the absorbance measurement can be performed within 10 min after the lid was opened.

### 3.2. Analytical Performance

Selected conditions were used for the evaluation of method performance. It was found that the calibration plot of ethanol concentrations in the range 1.0–90% (v/v) was linear with the corresponding absorbance (Figure 2(d)). The linear equation *A*_590nm_ = (9.4 × 10^−3^) *C*_ethanol, %(v/v)_ + (0.1 × 10^−3^) where *A*_590nm_ was absorbance measured at 590 nm and *C*_ethanol, %(v/v)_ was ethanol concentration in % (v/v), and correlation coefficient (*r*^2^) = 0.9941, indicating good method linearity. This linear range covered an ethanol concentration of 3.8–40% (v/v) in the target alcoholic beverage samples. It was found to be wider than those of the previous gas separation units, summarized in Table 1. Therefore, no additional step of dilution or preconcentration of the samples is required before analysis. Limit of detection (LOD), calculated as 3*SD*_blank_/*m*, and limit of quantification (LOQ), calculated as 10*SD*_blank_/*m*, were 0.3 and 1.0% (v/v), respectively, where *SD* was the standard deviation of 10 blank measurements and *m* was the slope of the calibration curve. The LOD and LOQ also supported sufficient performance of this proposed method to analyse the ethanol degree present in the target beverage samples.

Since the MBL-VP unit was hand-made and all steps were manually operated, precision was the most important factor to consider. Additionally, the temperature is the most influential parameter for ethanol vaporization, which could vary method's sensitivity and precision. It was found in the previous studies that higher method sensitivity was obtained when increasing temperature [32, 33, 38]. In this work, it was also found that absorbance of the collected acceptor solutions significantly increased when temperature of the room changed from 25 to 30°C (data not shown). Thus, temperature of the experimental room must be controlled. To be simple and convenient, all experiments were carried out in a temperature-controlled room (28 ± 1°C) in this study instead of using additional heating apparatus, which was found to give sufficient sensitivity for the target samples. Within-day precision, indicated by relative standard deviations (RSD) of absorbance obtained from 5 replicate measurements, was 6.6 and 4.5% for 5 and 40% (v/v) ethanol, respectively. For interday precision, slopes of calibration curves in the range of 5.0–40% (v/v) ethanol constructed in 5 consecutive days showed an RSD of 8.3%. These results indicated that the MBL-VP unit could be manually operated with satisfactory precision. Despite the fact that analysis time per sample of this proposed manual MBL-VP operation was longer than most of the flow-based systems (Table 1), the MBL-VP units can be easily fabricated using lower cost and be operated in parallel for analyses of many samples. Hence, the average analysis time per sample can be shorter if the parallel analyses are efficiently carried out. Additionally, the proposed method showed comparable performance, especially in linear range, to other simple and cost-effective techniques (Table 2). Therefore, the proposed MBL-VP unit can be used as an alternative detection and quantification tool of ethanol in various types of beverages with the benefit of no additional sample pretreatment and simple, low-cost, and easy-to-make gas separator unit.

### 3.3. Analysis of Ethanol in Beverages and Method Validation

The proposed method was applied to analyse ethanol content in 12 samples, including beer, wine, spirit, and cocktail. Most of the samples were clear but had a different color. The results in Table 3 indicated that all analysed ethanol concentrations in the samples by this work were not different from the declared values shown on the labels (*t*_cal_ = 0.26; *t*_crit_ = 2.20; *P*=0.05). Additionally, all results given by this proposed method agreed well with those obtained by the conventional distillation/pycnometer method (*t*_cal_ = 0.33; *t*_crit_ = 2.20; *P*=0.05). Therefore, the proposed method was accurate sufficiently for analysis of ethanol in alcoholic beverages. To investigate matrix effects, recovery of ethanol spiked in those beverage samples was studied, which was found in the range 89−109%. The recovery study indicated that various colors and other nonvolatile and volatile species present in the sample did not disturb the colorimetric analysis of ethanol using the proposed method.

## 4. Conclusions

In this work, a so-called membraneless vaporization (MBL-VP) unit was utilized for the colorimetric determination of ethanol in beverage samples. The unit was home-made using a reused glass bottle. The alcoholic beverage was graduated and directly injected into a sample vial hung from the lid of the glass bottle without dilution, preconcentration, or distillation using a disposable plastic syringe. With the assistance of magnetic stirring for 5 min, vaporization of ethanol occurred with subsequent diffusing in an acidic Cr_2_O_7_^2−^ acceptor that was prefilled in the glass bottle for further reducing Cr_2_O_7_^2−^ to Cr^3+^. The amount of Cr^3+^ corresponding to ethanol content in % (v/v) was measured using a UV/Vis spectrophotometer (590 nm). Under the best conditions, the proposed MBL-VP unit showed a wide quantification range covering the ethanol content in the target beverage samples with sufficient precision and had no effect from matrix. Accuracy of the proposed method was confirmed using the reference distillation/pycnometry method. The MBL-VP, as a homemade gas separator, was successfully applied to the analysis of ethanol in alcoholic beverages with the advantages of ease in making, simple and low-cost manual operation, and less energy consumption.

## Figures and Tables

**Table 1:** Some analytical performance of the proposed MBL-VP unit and the previous MGD formats.

Type of gas separator/operation mode	Chemistry/detection	Analytical performance				References
		Linear range (% v/v)	RSD (%)	LOD (% v/v)	Analysis time per sample (min)	
Membrane/FI	Cr_2_O_7_^2−^ oxidation/spectrophotometry	1–30	0.55	0.68	≈3	[36]
Membrane/FI	Ceric (IV)-ethanol complexation/spectrophotometry	0.1–10	<1.3	0.03	3	[27]
Membrane/FI	Enzymatic/spectrophotometry	0.05–0.5	<2.3	0.002	6	[28]
Membrane/FI	Enzymatic/amperometry	0.01–60	0.23–0.65	0.0006	≈0.3–0.5	[29]
Membrane/FI	Cr_2_O_7_^2−^ oxidation/spectrophotometry	Up to 50	<2	—	2	[52]
Membrane/FI	Cr_2_O_7_^2−^ oxidation/potentiometry	Covered the range 3–40 in the samples	0.8	—	2.4	[53]
Membrane/FI	Hypsochromic shift of absorption band of methyl orange/spectrophotometry	5–45	1.3	2.23	3	[54]
Pervaporation/FI	Cr_2_O_7_^2−^ oxidation/spectrophotometry	1–20	3	0.5	10	[33]
Pervaporation/FI	Cr_2_O_7_^2−^ oxidation/spectrophotometry	1–10	<1.5	—	3	[32]
Membraneless/FI	Cr_2_O_7_^2−^ oxidation/spectrophotometry	0.5–30	0.50	0.27	≈3.8	[36]
Membraneless/FI	Cr_2_O_7_^2−^ oxidation/spectrophotometry	5–30	3.7	2.68	2.4	[38]
Membraneless/FI	MnO_4_^−^ oxidation/spectrophotometry	5.0–15.0	0.24–0.92	0.26	2.5	[39]
Membraneless/manual	Cr_2_O_7_^2−^ oxidation/spectrophotometry	1.0–90	<10	0.3	≈5	This work

FI: flow injection analysis.

**Table 2:** Some analytical performance of the proposed MBL-VP unit and other simple and cost-effective approaches for quantification of ethanol in beverages.

Technique	Fabrication of platform or reaction vessel	Sample pretreatment/preparation	Chemistry/detection	Analytical performance				References
				Linear range (% v/v)	RSD (%)	LOD (% v/v)	Analysis time per sample (min)	
Lab-on-a-chip	Prepare PMMA mold using laser cutting ⟶ fabricate pattern on PDMS chip	Dilution	Ceric (IV)-ethanol complexation (performed on microchannel)/spectrophotometry	0.20–20	<1.40	0.039	≈1.3	[21]
Smartphone-based digital image	Make a tube holder from acrylonitrile butadiene styrene using 3D printing	No	Changing in resonance structure of phenolphthalein in alkali medium (performed in a microtube)/digital image	10–70	1.2	2.1	<1	[22]
Spot-test, smartphone-based digital image	Make a plastic chamber for photographing	No	Cr_2_O_7_^2−^ oxidation (performed in a porcelain plaque)/digital image	1–20 and 25–50	NR	0.25	12	[23]
Electrical conductometry	Connect a jacketed glass beaker containing a magnetic stirrer to a thermostat and conductivity meter	Not required, but limited to distilled samples	Uncatalyzed esterification between ethanol and acetic acid (performed in a jacketed glass beaker)/electrical conductometry	0.0–99.9	<3.45	0.63	5	[26]
Visible chemical wave (instrument free)	Prepare metal catalyst gel dish	Dilution	Belousov-Zhabotinsky reaction (performed in a catalyst gel dish)/visible chemical wave (using smartphone camera)	0.2–1.0	<2.9	NR	5	[24]
96-well-plate	Using commercial 96-well-plate	No	Changing in resonance structure of phenolphthalein in alkali medium (performed in test-tube before transferring to a 96-well-plate)/digital image using desktop scanner	33–45	1.8–2.6	NR	2.5	[25]
Diffusive microdistillation device	Using commercial 5 mL polypropylene tube to contain acceptor inserting in a 50 mL polypropylene tube containing a sample	Heating to 80°C	Cr_2_O_7_^2−^ oxidation/spectrophotometry	1–12	3.8	0.16	15	[51]
MBL-VP	Make an MBL-VP unit using a reused glass bottle as an acceptor container, and a plastic vial for holding a sample	No	Cr_2_O_7_^2−^ oxidation/spectrophotometry	1.0–90	8.3	0.3	≈5	This work

NR: not reported.

**Table 3:** Ethanol concentrations and their recoveries in beverage samples by the proposed MBL-VP unit in comparison with the conventional method.

Sample			Ethanol concentration (% v/v)			Recovery (%)
No.	Type	Appearance	Declared on label	Pycnometry (*n* = 1)	This work ± SD, (*n* = 3)	
1	Beer	Light brown	5.0	4.79	4.6 ± 0.6	109
2	Beer	Brown	6.5	6.45	6.3 ± 0.6	101
3	Beer	Light brown	5.5	5.76	5.6 ± 0.2	97
4	Wine	Red	7.0	7.63	8.5 ± 1.8	89
5	Wine	Red	12	12.28	13.0 ± 1.6	92
6	Spirit	Colorless	17.8	17.94	18.0 ± 2.9	97
7	Spirit	Dark brown	35	36.6	37.0 ± 1.8	96
8	Spirit	Colorless	7.0	7.47	8.1 ± 0.2	101
9	Spirit	Colorless	40	40.27	35.0 ± 0.7	89
10	Cocktail	Orange	3.8	3.78	4.1 ± 0.7	99
11	Cocktail	Light blue	5.0	5.23	5.8 ± 0.8	90
12	Cocktail	Light green	4.5	4.36	4.7 ± 0.7	101

## Data Availability

The data used to support the findings of this study are included in the article.
